# The generalized harmonic potential theorem in the presence of a time-varying magnetic field

**DOI:** 10.1038/srep35412

**Published:** 2016-10-17

**Authors:** Meng-Yun Lai, Xiao-Yin Pan

**Affiliations:** 1 Department of Physics, Ningbo University, Ningbo, 315211, China

## Abstract

We investigate the evolution of the many-body wave function of a quantum system with time-varying effective mass, confined by a harmonic potential with time-varying frequency in the presence of a uniform time-varying magnetic field, and perturbed by a time-dependent uniform electric field. It is found that the wave function is comprised of a phase factor times the solution to the unperturbed time-dependent Schrödinger equation with the latter being translated by a time-dependent value that satisfies the classical driven equation of motion. In other words, we generalize the harmonic potential theorem to the case when the effective mass, harmonic potential, and the external uniform magnetic field with arbitrary orientation are all time-varying. The results reduce to various special cases obtained in the literature, particulary to that of the harmonic potential theorem wave function when the effective mass and frequency are both static and the external magnetic field is absent.

The harmonic potential theorem (HPT)^1^ concerning the many-body system trapped in an external harmonic potential describes the evolution of the wave function (WF) under the influence of an arbitrary external uniform electric field. It plays a significant role in time-dependent (TD) phenomena of quantum many-body systems. In particular, in the TD theories such as the TD density functional theory^2^,^3^,^4^,^5^, TD quantal density functional theory^6^,^7^ and also in Bose-Einstein condensation(BEC)^8^. Soon after being discovered, the theorem was investigated in the presence of a static magnetic field but in the absence of harmonic potential by Vignale^9^, whereby he proved the theorem by the observation that an applied uniform TD electric field can be eliminated by transforming the static harmonically trapped system to an accelerated reference frame according to the classical equation of motion of the mass center. Furthermore, he stated that HPT is valid even in the case when the uniform magnetic field is time-varying(TV) and gave the explicit form of the TD potential in the accelerated frame. Recently, there has been considerable interest in the study of the theorem itself^10^. It has been generalized to the harmonic potential whose frequencies are TV^11^, the explicit form of the HPT WF in the presence of a perpendicular static magnetic field is also obtained^12^.

In the meantime, a TV effective mass could simulate the input or removal of energy from the system^13^. If quantum many-body systems interact with TV environment such as temperature, pressure, stress and energy, the effective masses will be modified. Thus, if the environment changes as time goes by, one would have a system with TV effective masses^14^. Thus, the study of harmonically trapped quantum systems with TV masses, particularly the model of TD harmonic oscillators (TDHO)^15^,^16^ has been extensively investigated (see, e.g.^17^,^18^,^19^,^20^,^21^,^22^,^23^,^24^,^25^,^26^,^27^,^28^,^29^). Moreover, there has been considerable interest in the quantum systems in a TV electromagnetic field^18^,^30^,^31^,^32^,^33^,^34^,^35^,^36^,^37^,^38^. The quantum problems that combine a TV effective mass and a TV external magnetic field have also been investigated. For instance, the Feynman’s propagator for a charged particle with TV mass in a TV magnetic or electromagnetic field were obtained^39^,^40^. Nevertheless, these studies all focused on the single-particle problems.

Besides its tremendous theoretical interest, the model of TDHO can be mapped to (i) a system of electric and magnetic fields in the interior of a Fabry-pérot cavity^41^,^42^,^43^, or (ii) in a cavity filled with a medium with TV dielectric constant^44^, or (iii) Paul traps systems^45^,^46^,^47^ where atomic particles are trapped in TV electromagnetic fields, or (iv) the effective Hamiltonian for dissipative systems^24^,^48^ confined in quantum dots^49^, if one applies TV electromagnetic fields to some of above mentioned systems, say the dissipative systems in quantum dots^50^, then one will obtain the model Hamiltonian (see eq. (1) below), or (v) a Bose-Einstein condensate subject to a rotating harmonic potential^51^ (see the Discussion below).

In light of above facts, in this work we try to investigate the HPT in the most general form, i.e. the case when the effective mass, confining frequencies, and the external uniform magnetic field are all TV. After stating the generalized HPT, we then give the proof via two different approaches, i.e. the operator method and the accelerated frame approach^9^. We show that the WF is still comprised of a phase factor times the solution to the unperturbed TD Schrödinger equation with the latter being translated by a TD value that satisfies the classical driven equation of motion.

## Results

### Hamiltonian and the generalized HPT

Consider a system of *N* identical particles with a TV effective mass *m* = *m*(*t*) under an external TV magnetic field with arbitrary orientation **B**(*t*) = (*B*_1_(*t*), *B*_2_(*t*), *B*_3_(*t*)), confined in an external TD harmonic potential 
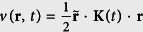
, with **K**(*t*) an symmetric positive 3 × 3 matrix. The TD harmonic potential can be used to describe many experimental situations. For instance in BEC experiments, it can describe rotating the quadratic trap^51^, modulated trapping^52^, or reflecting fact that the trap is perturbed to obtain the response spectrum of the condensate^53^,^54^,^55^. The two-body interaction between the particles *u*(**r**_*i*_ − **r**_*j*_) can be of arbitrary form. A uniform TD driving electric field **E**(*t*) = **f**(*t*)/*q* is turned on at time *t* = 0 with *q* = −*e* the charge of an electron. Thus, in the coordinate representation the Hamiltonian reads


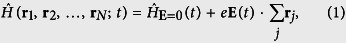


where the unperturbed component is


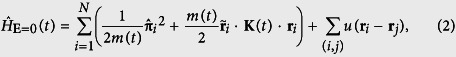


with 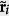 the transpose of the position vector **r**_*i*_. 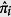 is the physical momentum operator


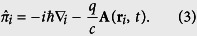


Choosing the symmetry gauge such that the vector potential **A**(**r**_*i*_, *t*) = (**B**(*t*) × **r**_*i*_)/2, and substituting eq. (3) into eq. (2) yields


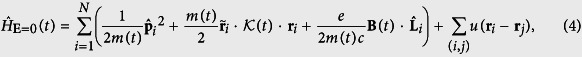


where 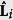 is the angular momentum operator of the *i*-th particle, and


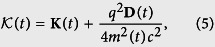


is still a 3 × 3 real positive symmetric matrix with


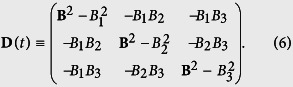


The model above considered is obvious an open system. Generally there exist two main approach for such systems. The first is so called system-plus-bath approach^56^,^57^,^58^, and the second one is the effective Hamiltonian approach^24^,^59^,^60^. The effective Hamiltonian usually has a TV mass that arises due to the interaction between the system and the bath^48^, and governed by the TD Schrödinger equation within the adiabatic approximation. Hence, the Hamiltonian of eq. (1) can be regarded as an effective Hamiltonian for some open system and obey the following TD Schrödinger equation,


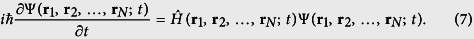


The core of the generalized HPT is the solution to the TD Schrödinger equation eq. (7). We refer to this solution as HPT WF. The generalized HPT states that the following WF


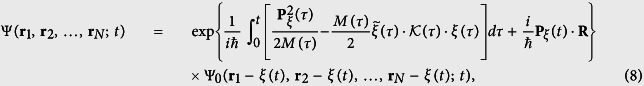


satisfies eq. (7), where Ψ_0_(**r**_1_, **r**_2_, …, **r**_*N*_; *t*) is the solution of the following unperturbed Schrödinger equation:


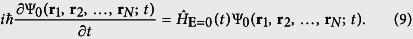


Note that the phase factor in eq. (8) has the form similar to classical action. In eq. (8), *M*(*t*) = *Nm*(*t*), *ξ*(*t*) is the translation vector, **P**_*ξ*_(*t*) the corresponding momentum vector (see eq. (26) below), and 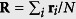 the center of mass coordinate. The translation vector *ξ*(*t*) satisfies the classical equation of motion


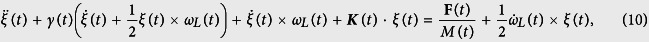


with 
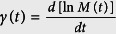
, *ω*_*L*_(*t*) = *Ne***B**(*t*)/(*M*(*t*)*c*) = *e***B**(*t*)/(*m*(*t*)*c*) the cyclotron frequency, and **F**(*t*) = *N***f**(*t*). Eq. (10) is just the classical equation of motion for a harmonically trapped particle with a TV mass in the presence of TV external magnetic field **B**(*t*), perturbed by an external force **F**(*t*).

### Proof of the theorem via derivation

Next we prove the generalized HPT by derivation. Using the center of mass (CM) and relative coordinates and momentums^61^,^62^,^63^


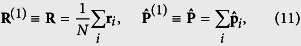


and


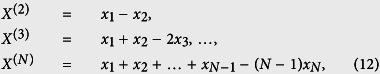


and similarly for *Y*^(2)^, …*Y*^(*N*)^, *Z*^(2)^, …*Z*^(*N*)^, and *P*^(2)^, …*P*^(*N*)^, the Hamiltonian of eq. (1) can be decomposed into the CM and relative motion parts,


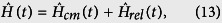


where


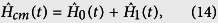


with


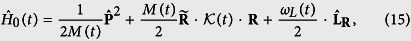


and


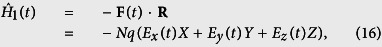


is the perturbation term due to the external electric field, and 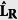 the angular momentum operator for the CM coordinate **R**. The relative motion part 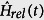 contains only the relative coordinates, hence 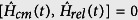. Consequently, the CM motion and the relative motion are separable. Therefore, the total WF of the Hamiltonian is the product of the WFs of CM motion and relative motions:


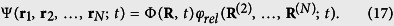


The relative motion WF 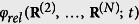 and the CM motion WF Φ(**R**, *t*) satisfy their own Schrödinger equations, respectively, with certain initial conditions. In the following, we shall focus on the CM motion Hamiltonian of eq. (14), and try to find its WF Φ(**R**, *t*). Similar to the structure of the HPT WF, let us assume


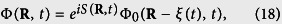


where Φ_0_(**R**, *t*) is the WF for the unperturbed CM motion Hamiltonian, i.e. which satisfies the following Schrödinger equation,


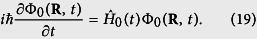


Next, we shall seek the analytical expressions for 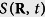 and determine the translated vector *ξ*(*t*) which leads Φ(**R**, *t*) to satisfy its own Schrödinger equation,


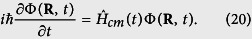


Inserting eq. (18) into eq. (20) and using eq. (14), we have


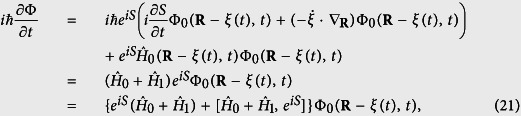


Note here have used that fact that 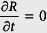 since *R* now is the eigenvalue of the coordinate operator 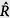, whose hat has been dropped since we work in the coordinate representation. With the ansatz that the phase factor can be cast into the following form,


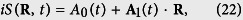


then we have the commutator


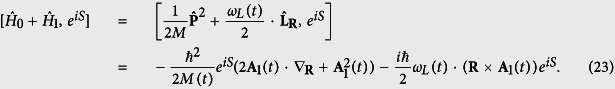


Making use of the following expression


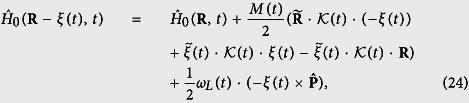


and inserting eqs (23) and (24) into eq. (21) yields


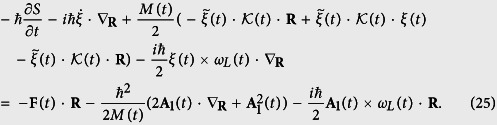


By comparing the coefficients of 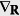 on both sided of eq. (25), we have


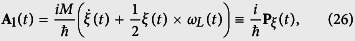


and similarly for the coefficients of **R**, we obtain


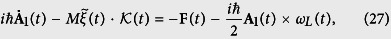


and


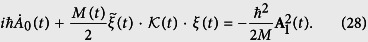


Inserting eq. (26) into eqs (27) and (28), we immediately find that the translation vector satisfies eq. (10). Note that 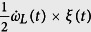 is the induced electric force by the TV magnetic field, and


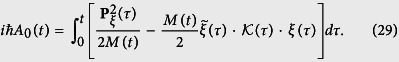


is just the classical action without the electric field term. Hence, from eqs (17), (18), (22), (10) and (29), we obtain the final WF of eq. (8). In other words, we have proved via derivation that the HPT WF eq. (8) is the solution of the TD Schrödinger equation eq. (7).

The HPT WF is the key result of this paper. Note that if one requires that the initial state is the eigenstate of the unperturbed Hamiltonian, i.e. Ψ(*t* = 0) = Ψ_0_(*t* = 0), then usually one has the initial conditions: *ξ*(0) = 0, 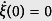. We stress that the HPT WF can reduce to various special cases existed in literature^1^,^9^,^10^,^11^,^12^. Thus, we have extended the HPT to the case when the quantum systems have a TV effective mass and TV confining frequencies, in the presence of a uniform TV magnetic field with arbitrary orientation.

### The Hamiltonian and wave function in the accelerated frame

Inspired by the method of Vignale^9^, we next show that our results can also be obtained by transforming the system to an accelerated reference frame. Making the acceleration transformations


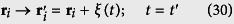


on the system with *ξ*(*t*) governed by eq. (10), hence the connection between the original WF Ψ(*t*) and the accelerating WF Ψ′(*t*) is


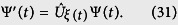


The WFs Ψ(*t*) and Ψ′(*t*) in the above equation satisfy the following Schrödinger equations respectively:


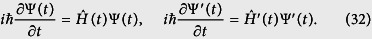


And the explicit form of the unitary operator 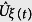 is^64^


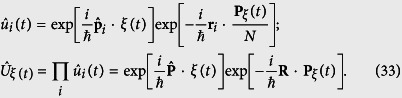


From eqs (8), the connection of the accelerating WF and the WF in the absence of the external electric field can be written in a simpler form as


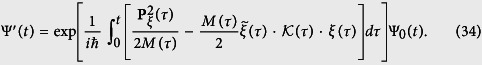


Applying 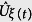 to the Schrödinger equation eq. (32), yields the explicit expression of the accelerating Hamiltonian,


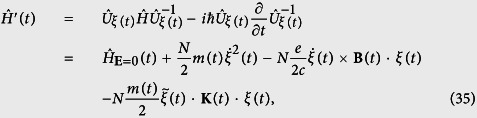


after a long calculation, above Hamiltonian can be recast into the following form


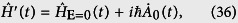


thus we immediately recognized that the WFs for 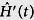 and 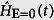 only differ by a phase which is exactly eq. (34). On the other hand, from eqs (34) and (36), one can readily see that the uniform time dependent electric field is eliminated by performing the acceleration transformations. This proves again the generalized harmonic potential theorem.

## Discussion

In summary, we have presented the detailed analytical form of the evolution of the WF for an quantum system with TV effective mass trapped in a harmonic potential with TV frequency, in the presence of a TV uniform magnetic field with arbitrary orientation, and driven by a TD **uniform** electric field. It is found that the WF is comprised of a phase factor times the solution to the unperturbed TD Schrödinger equation with the latter being translated by a TD value that satisfies the classical equation of motion for a driven harmonic oscillator with TV mass in the presence of an external TV magnetic field. The analytical form of the phase is also given. The results can reduce to various special cases existed in the literature. We also show that our results can be obtained by transforming the system to an accelerated frame. Moreover, we stress that our results are applicable to both the fermionic and bosonic systems with general effective masses and external magnetic fields that can be described by some smooth functions of time, since the derivations do not rely on the statistical properties of the WF or any specifically choice of the TD terms and parameters. However, the external TD electric field must be uniform.

Finally, we briefly discuss some real physical systems that our results might shed lights on. Notice that if one identifies the angular velocity 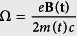 and the gravity with the external driving force, then the model Hamiltonian of eq. (1) can be used to describe atoms trapped in a harmonic potential rotating instantaneous around the *z* axis^51^, the related experiment has been done at ENS^65^. In above case of a vertical axis of rotation, the only effect of gravity is a displacement of the equilibrium position^66^ thus can be ignored. When the axis of rotation was titled away from the trap axis such those experiments done in refs 67,68, the effect of gravity must be taken into account. For instance, in a uniformly rotating trap, it can causes resonances hence the escape of the center of mass for a collection of interacting particles from the trap^66^. Our results implies that even the rotation is titled and TV, the effect of gravity is solely to transport rigidly the center of mass, or density distribution of the system. This is expected to be confirmed experimentally.

## Additional Information

**How to cite this article**: Lai, M.-Y. and Pan, X.-Y. The generalized harmonic potential theorem in the presence of a time-varying magnetic field. *Sci. Rep.* **6**, 35412; doi: 10.1038/srep35412 (2016).
